# Exploring willingness to participate in future Human Infection Studies in Lusaka, Zambia: A nested qualitative exploratory study

**DOI:** 10.1371/journal.pone.0254278

**Published:** 2021-07-09

**Authors:** Evelyn Muleba Kunda-Ngándu, Masuzyo Chirwa-Chobe, Chanda Mwamba, Jenala Chipungu, Esnart Ng’andu, Hope Mwanyungwi Chinganya, Michelo Simuyandi, Roma Chilengi, Anjali Sharma

**Affiliations:** Research Department, Centre for Infectious Disease Research in Zambia, Lusaka, Zambia; Makerere University, School of Public Health, UGANDA

## Abstract

Human Infection Studies (HIC) involve intentional infection of volunteers with a challenge agent or pathogen with the aim of understanding and developing vaccines as well as understanding the disease pathophysiology in a well-controlled environment. Though Africa carries the highest burden of vaccine-preventable diseases, the region is only now being primed to conduct HIC relevant to its population. Given the imminent introduction of HIC in Zambia, we sought to understand potential participants’ willingness to volunteer for such studies. We used a qualitative exploratory approach to understand the potential participants’ perceptions on willingness to participate in HIC using the example of typhoid. Healthy adults, recruited using random selection and purposive sampling from higher learning institutions in Lusaka, participated in 15 in-depth interviews (IDIs) and 5 Focus Group Discussions (FGDs) respectively. Participants considered typhoid a serious disease with potential for life-long consequences and death. After sharing audio-visual materials introducing the concepts of HIC, some participants expressed open willingness to participate or alternatively the need to consult parents and professors, and expressed fear of death and illness. Though willing to be quarantined for up to six months, participants expressed concerns regarding separation from family and duties, having insufficient information to decide, inadequate access to care, severe disease, life-long injury or side-effects, death, and vaccine failure. These concerns along with possibility of underlying conditions that compromise individual immunity, competing priorities, parental refusal, and distrust of study or vaccine efficacy could lead to refusal to participate. Reasons for willingness to participate included monetary compensation, altruism and being part of a team that comes up with a vaccine. Though afraid of deliberate typhoid infection, potential participants are willing to consider participation if given adequate information, time to consult trusted persons, compensation and assurance of adequate care.

## Introduction

Typhoid fever accounts for an estimated 128 000 deaths each year in Low and Middle Income Countries (LMICs) [1, 2] with children below the age of five and those going to school most affected [3]. Fecal-oral transmission of *Salmonella enterica* serotype Typhi (*S*. *typhi*) [4] causes approximately 12 million typhoid infections globally per year. While clean water, sanitation and hygiene (WASH) are sufficient to interrupt transmission, LMICs struggle to keep up with the increasing demand for WASH by a rapidly growing urban population [5]. Where governments have managed to put in infrastructure, prohibitive costs of installing water and sewage connections, absentee landlords, and lack of government subsidy prevent uptake by the urban poor [6]. However, even with fully functioning and connected infrastructure, WASH uptake may be sub-optimal if residents do not fully agree with biomedical explanations regarding cholera causation, prevention, and treatment [7]. Thus, vaccines remain the most viable short to medium term option for diarrhoeal disease control.

However, some vaccines developed in non-endemic countries have proven to be less effective in LMIC settings [8, 9] highlighting the urgent need to develop new effective vaccines and improve already existing ones among disease-endemic populations [8]. Human Infection Challenge Studies in endemic countries, therefore, would prove to be more efficient and effective in testing and developing novel vaccines. This is because there are more compelling ethical and scientific reasons to conduct HIC in LMICs, including but not limited to developing new interventions and improving health of local people [8, 10].

Though Africa carries the highest burden of vaccine-preventable diseases, it is only now that the region is beginning to conduct Human Infection Studies (HIC) relevant to its population. HIC can accelerate vaccine development by facilitating an understanding of pathophysiology and mechanics of immune response under highly controlled conditions and to efficiently test vaccine or drug efficacy. However, the intentional infection of healthy study participants with a viable challenge agent or pathogen albeit under highly controlled conditions [8], could potentially raise public concerns, queries, and ethical complexities particular to LMIC settings. These may include concerns around communal pressure to participate, local language wording in information sheets, beliefs on blood collection, adequacy of infection control measures in the community, and appropriateness of compensation given levels of poverty [11]. Additionally, the daily risk posed in some LMIC environments makes it difficult to control HIC participants’ exposure to additional diseases or the spread of infection into the larger community without residency requirements [9, 11]. In times where there is no apparent treatment or rescue for a selected pathogen, it must be clear that benefits outweigh the risks as maybe the case for COVID19 [12]. However, there is evidence that HIC can be conducted without serious adverse events or hospitalizations [13] making undertaking HIC in LMICs even with limited access to healthcare ethically permissible. Furthermore, there is evidence that lack of medicines and other supplies, not only impede delivery but decrease access to health care [14]. Gathering public opinion of HIC including ethical and scientific concerns among potential participants is an important step in establishing a platform to conduct HIC in LMICs.

Given the imminent introduction of enteric HIC in Zambia, we conducted a qualitative exploratory study nested within an immunological study that compared immune responses to natural typhoid infection amongst Zambian patients, and to deliberate typhoid infection amongst British volunteers. In our nested qualitative study, we explored willingness of potential participants to participating in a HIC. We also explored knowledge regarding typhoid and perceived risk as these elements could influence willingness to participate in HIC among students from institutions of higher learning. Additionally, we explored knowledge of HIC, and reactions when introduced to the concept behind HIC among potential participants in Lusaka, Zambia. Gathering would-be participants’ perspectives can aid the process of developing practical and ethical guidance cognizant of societal values and constructions of risk around enteric HIC [10, 15]. Extending these findings to further public engagement activities can build and enhance study accountability to the community being served [11].

## Methods and materials

### Study area

The study was conducted in areas surrounding the University Teaching Hospitals (UTH) in Lusaka, the capital city of Zambia. The UTH is Zambia’s largest referral hospital serving a population of over 2 million people with cases referred from all provinces of Zambia [16]. The qualitative aspect of the study was done at universities namely, University of Lusaka and the University of Zambia and colleagues namely, National Institute for Public Administration and The Lusaka Business Colleges.

### Study respondents

We conducted 15 in-depth interviews (IDIs) and 5 focus group discussions (FGDs) as shown in Table 1, among healthy adults recruited from higher learning institutions. Inclusion criteria for study participation for both IDIs and FGDs included being an adult aged 18 or above, ability to provide informed consent and willingness to participate in all study procedures including blood sampling. The exclusion criterion was any condition that renders an individual incapable of providing consent.

**Table 1:** Study participants.

IDIs	Total no. of participants	No. of female participants	No. of male participants
**Total. 15**	15	3	12
**FGDs**			
No. 1	8	5	3
No. 2	11	11	0
No. 3	10	10	0
No. 4	10	0	10
No. 5	12	3	9

The study was conducted among students from two universities and three colleges. We conducted both IDIs and FDGs for an in-depth exploration of the data. In-depth interviews were conducted for a more one-on-one interaction, while FDGs were conducted to gather more information at a faster rate while drawing upon the respondents’ reactions and experiences. IDI participants were randomly selected from among medical and nursing students at the University of Zambia School of medicine and the Lusaka School of Nursing. The sampling frame consisted of all student rooms in both universities, which were selected using a script written in Python 3.6 for a simple random sampling. Study staff went to selected rooms and all occupants were invited to learn more about the study at the UTH research site. All occupants present at the time of the visit and willing to participate were invited to the research site. Consenting individuals participated in the in-depth interviews at the study site within the UTH.

Participants for the FGDs were sampled purposively to include non-medical/nursing students from the University of Lusaka, the Lusaka Business and Technical College, and the National Institute of Public Administration. Each multidisciplinary group had at least eight students. This was done to have a more representative sample, with a more diverse reach in terms of profession. It would be assumed that medical students are more likely to know about HIC than non-medical students, therefore having most professions represented provides a balance and a true representation of the views explored. Three groups were single-sex, and two were mixed-sex groups. Study staff stationed at the selected higher institutions of learning invited students to learn more about the study using sensitization talks. Potential participants meeting the study eligibility criteria were scheduled to return on a specific day for consenting procedures and group discussions. Study staff provided detailed sensitization to all returning participants and administered informed consent procedures. To ensure participant confidentiality and encourage interactive participation, FGDs were conducted in private rooms.

### Study procedures

After providing written informed consent, a blood sample was collected from all IDI and FGD participants. 5ml of blood was collected via venipuncture into EDTA bottles on scheduled interview days. Samples collected were tested for typhoid specific antibodies to explore objectives of the quantitative arm of the study including immune profiling to establish history of previous typhoid exposure in otherwise healthy volunteers. Data for the quantitative arm is under analysis and will be published as a separate paper. For the qualitative arm, after collecting blood samples, participants were given time to settle in time for the FGDs. The participants were welcomed to the discussion and were advised to be free as they expressed their views.

### Data collection

IDIs and FGDs were conducted in the English language with written informed consent including for audio-recording to ensure that information was accurately captured. Trained study staff asked participants questions related to typhoid transmission, knowledge of HIC and, if they would participate or allow a relative to take part in a HIC. Trained study staffs took notes to supplement the recorded data. After determining what participants knew about HIC, they were shown a video on HIC [17] to allow them to make a more resolute decision of whether or not they would participate or allow participation in a HIC.

### Interview instruments

In-depth interview guides and Focus group discussion guides were used to explore participant knowledge of typhoid, HIC and perceptions surrounding participation in HIC. See Table 2 as an example of the questions in the guides.

**Table 2:** Questions and initial themes.

Topic	Questions	Rationale
**Typhoid knowledge/attitude**	1. Could you please tell us what you know about Typhoid fever?	Beliefs about typhoid and typhoid treatment
	2. How does a person get infected with typhoid fever?	
	3. How is Typhoid spread?	
	4. What are your views about people who get Typhoid?	
	5. Can you tell us about an experience with a family member that had Typhoid? [You do not have to mention your relationship with this person]	
	6. How were these family members cared for when they were sick?	
	7. How do people behave towards people suffering from typhoid?	
	8. What traditional remedies do you know of for the treatment of typhoid?	
Typhoid Human Challenge Trials	1. Have you ever heard of them?	To understand the general knowledge on the subject of HIC
	**If yes,** what do you know about Human Infection Challenges?	
	*Show video recorded vignette on human challenge trials*	
	2. What have you learnt from the video you have just watched?	
	• What parts of the video were confusing? What parts didn’t you understand? (Interviewer explains video to ensure that participants understand the video).	
Views on Participating in a HIC Trial?	1. How would you feel about being a participant in this type of study?	To get an understanding of whether or not the participants would participate in a HIC as well as explore the possible concerns including from family members and communities they come from
	a) Why do you feel this way?	
	2. What factors do you need to think about before deciding to participate?	
	3. How would you allow members of your family to participate in the trial? If not why not?	
	a) What concerns do you have on husbands participating in HIC trials?	
	b) What concerns do you have on wives participating in HIC trials?	
	c) What concerns do you have on children aged 18years and above participating in HIC trials?	
	4. What are your views on the extended time participants would have to spend away from their families if they agreed to be part of the study?	
	a) What implications would this have on:	
	i. Family	
	ii. Relationships	
	iii. Community	
	5. What are your thoughts around people being infected with typhoid for the purpose of gaining new knowledge that will contribute to the development of effective typhoid vaccination?	
	a) What issues does someone need to consider before accepting participate in a HIC trial?	
	6. If you decided to participate in a HIC trail, how would your family and community benefit from your participation?	
	7. What do you feel should be done to adequately prepare someone to participate in a HIC trial?	
	**1.** How do you feel about being a participant in this type of study? What has made you feel this way?	
	a) What fears and concerns would you have around participating in this study?	
	b) Who would you need to consult in order to decide to take part in the trial? Why this person?	
	c) What do you think this person can say about you taking part in the study?	
	d) How long do you think it can take you to decide to take part in this study?	
	e) What is the maximum length of time you can be isolated from your family?	
	f) Would you participate in this type of study?	
	** If your answer is “No”:**	
	g) Please tell me why you would choose not to participate?	
	Probe:	
	○ Family?	
	○ Friends?	
	○ Fear of infection?	
	○ Fear of death?	
	○ Work?	
	h) What can convince you to change your mind about participating in the study?	
	i) If you were offered a compensation for participating in the study, how would this affect your decision to participate? (Skip to section D).	
	** If your answer is “Yes”:**	
	j) What is your motivation for deciding to participate in such a study?	
	k) For what reason would you change your mind on participating in such a study?	
	l) How do you feel about being deliberately infected with typhoid?	
	m) How do you think you would be treated by your family and friends if they know that you have been infected with typhoid?	
	n) Once you agree to take part in the study, you will need to be confined for a period of time. What conditions would the study need to meet to ensure that you’re comfortable during the time you’re enrolled in the study?	
	○ What type of place would you be comfortable staying in?	
	○ How would it make you feel about not seeing your family for an extended period of time?	
	○ How would your participation in this study affect your family/friends?	
	**Probe:**	
	• Emotionally?	
	• Financially?	
	• Socially?	
	• Routine?	
Community Perceptions	1. What would you tell your friends/neighbors about your family member’s absence?	To explore issues of participant participation in line with the community views and concerns
	2. Who would you choose to tell this to?	
	3. How do you think they can react?	
	4. If a neighbor health committee member asked about the absence of your family member, what would you tell them?	
	5. What reasons would some of your community members have against participating in human infection challenges?	
	a. Who would these people be?	
Compensation	1. What would you need to be compensated for if you participated in a human infection challenge?	To understand the issues with regard to compensation and the participation
	2. How much do you feel is a suitable compensation for you if you participated in a human infection challenge?	
	○ How would you break down this amount in relation to the things you’d need to be compensated for?	
	3. Who would you give to manage this money?	

### Data analysis

Thematic analysis was conducted to identify the themes emerging with regard to willingness of the students on participation in a HIC. All interviews were audio recorded using a digital recorder and later transcribed verbatim by the research team. The initial step in analyzing data was the development of codes. The first author EKN developed initial codes after reading the transcripts several times to develop a sense of the whole data. The codes were shared with the authors CM and AN, who are social and behavioral scientists and qualitative experts for review. The data analysts listened to the audio recordings and read the accompanying transcripts repeatedly to familiarize themselves with the data.

Using the Thematic Content Analysis (TCA) approach [18], all transcripts were loaded in Nvivo and read several times applying the deductive codes and identifying possible codes in the data inductively [19–21]. Similar codes emerging from the data were then merged to the deductive codes to create a codebook. Each code in the codebook was defined to ensure consistency in applying the codes. This was an iterative process through which codes were named and redefined accordingly. All the scripts were then coded and the process took about a 3 months period. S1 Codebook, provides a sample of the code book.

After the coding process was competed, all codes with similar concepts were grouped into themes and sub-themes for comparisons among concepts and perspectives for further exploration and interpretation of the data. The data analysis and review continued for a period of 8 months, and was refined to make the paper more replicable. The authors separately reviewed codes by analytically linking the presented themes to the set of transcripts to come to the final code book. Having agreed on the codes, the coding process, involving matching the codes with parts of textual data as illustrative of the code, codes were then grouped into categories and consequently themes. All the authors participated in this process of re-grouping of the codes, for the final presentation of the themes as reflected in Table 3.

**Table 3:** Analysis process (codes, themes and sub-themes).

Textual data	Codes	Theme	Sub-themes
*“So typhoid is a bacterial infection*. *It’s caused by salmonella typhi and it is spread by the oral fecal route*. *Yeah*, *it can cause diarrhea*, *fever and the like*, *yes… People get it when they put something contaminated with salmonella in the mouth*.*”(IDIp13*, *medical student)*	Knowledge on typhoid Perception/thoughts of typhoid patients	General participants’ perceptions of Typhoid	
	Family typhoid experience Care for a typhoid patient		
	Traditional remedies		
*“Really I don’t have much knowledge on them [HIC] but I have heard about them*.*”(IDIp9*: *Medical student)*	Knowledge on HIC	Prior knowledge of Human Infection Studies	
*“For me actually I would feel motivated to see my child (adult child)–since I am the father myself—to see my child who is participating in such program*. *I would look at it as my child being educated in that perspective or that field and my child would bring back that knowledge back into the house and not only my house but to the community as well which will bring change*.*” (FGD#4p3*:*non-medical student)*	Participating in HIC	Factors which may affect willingness to participate in a HIC	
	Consultation		
	Isolation		
	Perceptions on family members participating		
*… “But if it were up to me I think I will do it for the money … for the money … ye”… (FGD#2p5*:*non-medical student)*	Motivation for participation	Motivations for participating in a HIC	Monetary compensation
	Benefits for the community		Altruism—for the love of others
			Patriotism
*“… So I think my family would benefit; if the vaccine works and one of them get sick*, *it would help them*. *And if they compensate me by giving me a lot of money and some of them [family members] would go to school and get nice things–then that would be good …” (FGD#2p3*:*non-medical)*		Perceived community concerns and benefits	
*“… Like she is saying that you have to be fully honest with the patient with the things they are going to go through as you are doing the trial…” (FGD#2p5*:*non-medical)*	Fears and concerns of participation Expectations/Recommendations Kind of research facility	What to consider when contemplating formulating a HIC	Information provided to aid decision
			Assurance of provision for health care in the event of illness Perceived risk and burden of participation
			Unattractive features of residential stay
*“…Yes*, *because you might find that the staff are just junior trainees–we don’t want that because this is life we are dealing with*. *We want qualified people who can handle the issues at hand and I would want them to talk to me one-on-one*, *to take me through each step…” (FGD#4p1*:*non-medical)*			

### Ethical approval

The study was approved by the University of Zambia Biomedical Research Ethics Committee (Ref: 010-09-18). The National Health Research Authority authorized the conduct of the study. Selected institutions of higher learning provided permission to conduct the study among students. Written informed consent was provided by all participants prior to initiation of study procedures.

## Results

A total of 15 participants, three women (20%) and 12 men (80%) with an average age of approximately 28 years participated in the IDIs. Focus group discussants included 22 men and 29 women with an average age of 22 years.

Participants were enrolled in different programs, which included certificate, diploma, or degree programs, in development studies, education, business and entrepreneurship, information technology, law as well as medicine, public health, and other health sciences.

## General participants’ perceptions of typhoid

Many participants knew about typhoid fever with a few confusing it for other diseases such as bilharzia. Participants who had knowledge about typhoid knew that is spread through an oral fecal route and one needed to have ingested the bacteria Salmonella Typhi to get the infection:

*“So typhoid is a bacterial infection*. *It’s caused by salmonella typhi and it is spread by the oral fecal route*. *Yeah*, *it can cause diarrhea*, *fever and the like*, *yes*… *People get it when they put something contaminated with salmonella in the mouth*.*”*

***(IDIp13*, *medical student)***

Those less knowledgeable thought that typhoid could be transmitted by contact with fluid or faecal matter from a person who has typhoid.

Participants considered typhoid a serious disease with potential for life-long consequences and death. They thought that people, particularly children of low socio-economic status living in high density areas with no proper sanitation and no access to clean water were at risk of typhoid as below:

*“I think the majority of those infected are children*. *Other than the fact that they are children and their immune systems are not yet developed*, *they are also prone to it because children lack knowledge on hygiene to be able to say ‘this is dirty’ and ‘this is what*.*’ That’s why I feel like it is more common in children who have naturally low immune systems*. *Also*, *people who have low economic status are prone to poor hygiene*.*”*

***(IDIp14*: *Medical student)***

*“Okay*, *so mostly aah*, *probably from my reading*, *it’s usually gotten from places that are highly populated with very poor sanitary conditions especially in Africa because we are economically challenged*. *So you look at countries or areas that are economically challenged with large population and then with poor sanitation*. *You also find it usually among children because they play in dirty environments*. *So whenever I think of typhoid or a person with typhoid*, *I start picturing them living in an area which is financially challenged with poor sanitation*, *yes*, *and all that*.*”*

***(IDIp10*: *Medical student)***

## Prior knowledge of Human Infection Challenge Studies

Many participants had limited knowledge about HIC until they watched the video on HIC and its requirements. While medical students had some idea about HIC, others knew nothing at all about it which is to be expected given the newness of HIC implementation in LMICs as illustrated below:

*“Really I don’t have much knowledge on them [HIC] but I have heard about them*.*”*

***(IDIp9*: *Medical student)***

***“****I have never heard about it*.*”*

***(IDIp8*: *Medical student)***

*“I think the main focus is coming up with vaccines and they are looking at coming up with vaccines that will help people in the future*…

***(FGD1p1*:*non-medical student)***

## Factors which may affect willingness to participate in a HIC

Once educated on typhoid HIC using audio-visual materials, some participants expressed unrestricted willingness to participate. Some participants were so eager that they wanted precise information on when the studies will begin while others insisted we contact them when these studies commence so that they can volunteer in them. Under this broad category of willingness to participate in a HIC, the theme of motivation emerged along with sub-themes related to monetary compensation, altruism, patriotism, and perceived community and family benefits. The other theme that emerged was of ‘other considerations when contemplating participation’, with sub-themes such as information to aid decision making, assurance of provision for health care in the event of illness, and perceived risk and burden of participation. Participants also understood that with some HIC there may be need to be kept in-residence for a few weeks at a study facility to ensure that they are cleared of any infection [11].

Some participants said that they would look at the pros and cons of participating in a HIC and the experience and expertise of the research team because it affects their safety.

Participants had various views concerning allowing family members to take part in a HIC sharing similar concerns regardless of relationship to the family member. Some said that they would allow participation because family members have free-will and they are free to make their own decisions if they are informed. One participant said they would be proud if a child took part in such a study and that they would allow them to participate because it would not only benefit the family but the community as well.

*“For me actually I would feel motivated to see my child (adult child)–since I am the father myself—to see my child who is participating in such program*. *I would look at it as my child being educated in that perspective or that field and my child would bring back that knowledge back into the house and not only my house but to the community as well which will bring change*.*”*

***(FGD#4p3*:*non-medical student)***

Others said they would deny the family members permission because they would be scared of not knowing what will happen at the end of the study, either positive or negative. Some said that they would need to get more information in order to decide whether or not to allow them to take part as below:

*“I would have a lot of questions to ask and some level of uncertainty deciding to take part in the Human Infection Challenge Study*. *And I would like to find out more about what the organization conducting the research is all about*. *Personally I would be a bit nervous about it before finding out what the organization doing research is all about*.*”*

***(FGD#5p6*: *non-medical student)***

When asked about the concerns they would have if husbands, wives, and children above 18 had to take part, they stated various concerns including damage to their relationship since they would be far apart. One participant said that,

*“Relations will be put on hold*, *because distance and silence kills a relationship*”

**(*FGD#4p4*: *non-medical student).***

Some participants aired concerns about the couple’s sex life after the participant returns home as the partner may fear that the participant is still infected and could infect them during sexual intercourse. Some men said that they would not allow their wives to participate because the wife has to cook and take care of the children at home.

## Motivations for participating in a HIC

Participants said that they would be motivated to participate for the financial benefit, the love for others and to be part of a team that developed a new vaccine. Other than personal benefits, participants anticipated community and family benefits which motivated them to participate.

### Monetary compensation

The participants clearly stated that they would participate for the monetary compensation given for the risk, side effects, and time lost during study participation. When asked about who should manage the money they receive as compensation, some participants thought that their parents, siblings, and grandparents would be best suited to manage the funds because they would look at the big picture and invest it. Others said they would manage it themselves given that they are the ones who put their lives at risk and bore the pain and discomforts of being in the study.

*…“You know everyone in this world thinks about finances and all that*, *yeah*?*”*…

***(IDIp12*: *medical student)***

***…*** *“But if it were up to me I think I will do it for the money … for the money … ye”*…

***(FGD#2p5*:*non-medical student)***

One participant said he would give the money to an orphanage if after the trial he does not get sick because he would be getting money for free as below:

***“****I would give to an orphanage*. *If I am not sick and they give money*, *it is like they have given it [money] for free because I didn’t get sick and I am fine*. *Then why will I use that money for nothing when there are people in need of that same amount?”*

***(FGD#5p3*:*non-medical).***

### Altruism—For the love of others

Some participants anticipated that while they may not directly benefit from participating, their loved ones would gain from a new and more effective vaccine which motivated them. They said that they would participate for the love they had for others.

***“…*** *Okay*, *it feels good because at least I’m volunteering*, *I’m bringing up for the future generation*. *I might not benefit right now but it’s for the greater masses out there … I am a driving tool coming up with ways which at the end of the day*, *will actually save lives…”*

***(IDIp15*: *medical student)***

One participant said she would participate because of her experience when she visited her family in the Congo where she saw people were dying of Ebola. She did not want to imagine other people dying when a solution like a vaccine can be provided:

*“… Yes I was against it [the study on Ebola vaccine]*. *Right now*, *the disease [Ebola] is in my home town*. *In my province*, *people are dying and if they tell me to try it*, *I will do it*, *I will try it*. *I have seen people die*. *A husband dies and gives it to the wife*, *who passes it to the son and it goes on passing from one to another in the family*. *So if they bring it [HIC] and say*, *“Let us try it on you; we inject you with Ebola and then try the cure on you and see if it will work*,*” I will do it because I know it is a bad disease*. *Even if I know that there is an available cure*, *I want to try the new one and if it doesn’t work*, *I will go for the standard one*. *Yes*, *that is what I would do*…”

***(FGD#2p12*:*non-medical)***

It must be noted that, participants were assured that we would not undertake a HIC using pathogens that have no cure like Ebola and HIV.

### Patriotism

Participants were excited to volunteer in a HIC to contribute to science and be part of a team that comes up with a vaccine in Zambia. Interestingly, some participants said they would participate in HIC for the betterment of (health) research which would put Zambia on the global map.

*“… I will do it for the research*. *Meaning it is something new; it has never happened in Zambia*, *right*?! *So*, *it will market the country; like now*, *they are now producing their own thing*. *So*, *I will do it to try it out…”*

***(FGD#2p5*:*non-medical)***

*“… One reason specifically is that I want to be part of the team that can make a difference in changing the current catastrophe of typhoid*. *So it’s not just about having knowledge about it but also be part of a team that finds solutions to typhoid …”*

***(IDIp14*: *medical student)***

## Perceived community concerns and benefits

The theme discusses the concerns from the community and the family members and what they stand to benefit from the participation of the would-be participants.

Some participants indicated that monetary benefit to their families would drive them to take part in a study even if the family was worried for their health and possible death as below:

*“… So I think my family would benefit; if the vaccine works and one of them get sick*, *it would help them*. *And if they compensate me by giving me a lot of money and some of them [family members] would go to school and get nice things–then that would be good …”*

***(FGD#2p3*:*non-medical)***

“… *But obviously their concern would be*, *“Are you going to survive*, *is it safe*?*”…”*

***(FGD#2p2*:*non-medical)***

Other participants said they did not care what the community had to say regarding their participation with some specifying that the vaccine, if developed, would be of benefit to their community so it was worth volunteering even if the vaccine does not work. For example, one female discussant said that:

*“…I think if the vaccine works when they try it on me*, *they will make more and bring it to the community who will benefit because there will be less typhoid*. *But if it doesn’t work that means nothing [bad as there is no ready alternative anyway]*.*”*

***(FGD#3p1*:*non-medical)***

Following the above stated opinions form relatives to the participants, implications of relatives opinions are discussed further in the following section ‘other considerations when contemplating participation in HIC.

## What to consider when contemplating setting a HIC

Several other factors influenced decision-making with regard to HIC such as the adequacy of information provided to an individual who would like to participate, risk-benefit ratio, and what relatives have to say about their volunteering for a HIC.

### Information provided to aid decision

Participants said that they needed sufficient details about the study for them to make an informed decision. They said it was imperative that participants of a study fully comprehended the study procedures and the risks involved or anticipated. Hence, study personnel need to be honest and practice full disclosure so that volunteers know for what exactly they are signing-up. The participants suggested that providing this information would prepare the person mentally and that it was important for their sanity and wellbeing during the time they are enrolled:

*“There should be an education or something like that where people are informed and taught for them to be willing to participate*. *Because people can’t just come and they are told we have to try it on you*. *If they are more informed about it*, *they will be willing to do it*.*”*

(***FGD#2p10*:*non-medical)***

***“…*** *Like she is saying that you have to be fully honest with the patient with the things they are going to go through as you are doing the trial…”*

***(FGD#2p5*:*non-medical)***

### Assurance of provision for health care in the event of illness

The other consideration was the kind of health care that would be assured to the participants during such studies. Participation was contingent on having study personnel who are well trained to give them the required care in the event that they fall sick:

*“…Yes*, *because you might find that the staff are just junior trainees–we don’t want that because this is life we are dealing with*. *We want qualified people who can handle the issues at hand and I would want them to talk to me one-on-one*, *to take me through each step…”*

***(FGD#4p1*:*non-medical)***

### Perceived risk and burden of participation

Participants argued that because HIC is used to evaluate candidate vaccines, it was to a certain extent not safe. They perceived themselves at risk just by virtue of participation and were scared of dying should there be a failure of standard treatment (rescue). They expressed concern about the side effects and adverse events including death as a result of the various study procedures including the trial vaccine itself:

*“…the only concern is the possibility of having an infection in the time of isolation and if the drug really works [i*.*e to clear the developed infection]…”*

***(IDIp6*:*medical student)***

For some participants, potential concerns and fears were alleviated by the fact that the disease in question was curable and said it would have been different if the HIC was, for instance about HIV, which is not curable:

*“… Because that is not aggressive like HIV or Ebola*. *Typhoid is a small disease*. *They can do it because it can be cured; it is not like when you have it you can pass away directly*. *They can cure it*. *So*, *if it is for a good purpose*, *we would accept to participate*… *It is like malaria*. *(Fellow participants laugh) Yes—malaria is bad but there is actually a cure for malaria*. *It is not like HIV when you have it you don’t get cured*. *So if anything goes wrong*, *I will go to other doctors and they will provide medicine for me*. *(All Laugh) Yes…”*

***(FGD#2p12*:*non-medical)***

### Features of the research facility

Some other considerations had to do with leaving home or school and being confined to a study facility for reasons of infection control and disease monitoring. Participants said that it had to be worthwhile to stay in such a place away from family. They wondered whether or not the facility would have social amenities or entertainment. They asked questions such as, “Will we be allowed to have visitors or to see our loved ones?” Some participants also preferred a facility which does not look like a hospital or hospital ward but was more homely and comfortable:

*“… They will have to let me be free to move around … I have to be watching movies*, *I have to be doing all that … (laughs) yeah*, *at least so that I wouldn’t be bored …”*

***(IDIp5*:*medical student)***

*“… It shouldn’t be in any way related to how the wards are (hospital ward) where you are very congested … It should be a decent place*, *where someone can sleep*, *the room should at least have a TV for people to watch … At least food should be there*, *it should be a good place …”*

***(IDIp15*:*medical student)***

## Discussion

This study revealed that participation in a HIC for some well-educated would-be participant in Zambia was almost guaranteed if expectations such as provision of adequate information and assurance of safety were met. Participants were willing to take part as long as they were provided with adequate information to make an informed decision. Other participants indicated that not only would they participate for their own sake and monetary compensation but also for the love of others and to contribute to science, motives also expressed by studies in other LMIC settings [22, 23].

Participants emphasized that for an individual to participate, they needed to be provided with adequate information to make an informed decision. They stated that information provided should include the level of discomfort, the possible risks, and benefits to them as an individual. While these are standard components of an information sheet, experiences elsewhere highlight the need to inform participants that there may be no direct benefit to them from their participation rather the benefit is at community level through scientific innovation and enhanced public health [11, 24, 25]. Furthermore, the participants need to be informed that during the study period including, if they withdraw from the HIC, ongoing confinement may be necessary to ensure participant and community safety [26].

Information requirements for HIC are complex and long and thus most researchers target educated participants [11] bringing its own ethical issue of representation and unjust exclusion of those not educated [8]. Researchers must therefore devise ways in which participants from all walks of life, regardless of education, can access HIC information in a simple and precise way and can ask questions in myriad ways to enhance retention of information and comprehension [11, 24, 27]. A study conducted by Jamrozick and Selgelid [28], found that among the unresolved issues was the recruitment and payment of research participants. Among the questions raised where for instance, “conditions under which HICs should recruit students and/or highly educated individuals on the one hand, it has been argued that educated individuals are better able to give informed consent; on the other hand, students may feel pressure to participate which could undermine consent and, furthermore, excluding less well-educated individuals may be unfair and/or reduce the generalizability of HICs results to key populations” [28]. We suggest that as discussed earlier that information about HICs is unpacked to be understood even by lay persons and not necessarily include only those educated. Moreover, education is not a measure of understanding in itself.

Even with full information, some participants said they would only participate if they were granted permission from significant others raising questions of autonomy and individual consent. In such circumstances, “group informed consenting processes to complement individual sessions” may be necessary [23]. This is where participants are met as a group and study procedures are explained in detail and those who want to take part sign the consent form individually. However, this does not mean that providing consent after consulting significant others is problematic. Like, in cases of shared decision making [29], what matters is that a participant understands what it means for them to give consent to a particular study or procedure, more so, if asking others for their opinion aids their understanding and comfortability. This is so that individuals’ confidence in the processes of a given study is boosted.

Some participants reported that they needed to understand the level of risk that is involved in HIC for them to decide whether or not to take part. This is similar to studies done by Stunkel, who argue that volunteers considered risk before participation in a study [30]. If risk is not clearly stated by researchers and understood by the possible participant, recruitment maybe affected as people may resist participation due to unnamed and unnecessary fears. Thus, all fears that are attached to participating in HIC need to be addressed during sensitizations, engagement and study participation to improve understanding and ease recruitment and retention. Also, because of the influence of significant others on individual decision-making, risk communication during sensitization and community engagement is important to ensure community-level comprehension [31].

For some pathogens such as typhoid, participants may need to reside in a study facility, equipped with provisions to be closely monitored for symptoms or reactions to drugs or the pathogen to ensure early and effective treatment [11, 24]. Infection control measures at the facility further contain the pathogen thus preventing and protecting third parties from getting unwanted infections. Similar to requirements reported from Kenya [32], facilities need to cater to more than medical concerns to include comfort, entertainment, family visitations (if possible), and provision to meet pressing worldly obligations such as examinations and funerals. These provisions bring with them considerations of measures that protect participants from infections carried by visitors and the visitors, infection from the facility [8]. Additionally, women may have other requirements such as for menstrual hygiene management, contraception, and a room-mate to feel safe.

Among the various motives for HIC participation, monetary compensation was expressed highly suggesting the need to establish what is fair compensation in the Zambian context, a matter we will discuss in-depth in another publication. However, desire for monetary compensation did not preclude volunteerism. Some participants also expressed altruism, patriotism, and perceived community and family members’ benefits as motivations for HIC participation. Similar to other studies, it is evident that people do have altruistic reasons for taking part in clinical trials including HIC, being motivated to contribute to science or the health of other people [30, 33, 34]. Interestingly, Kraft et al., found that some participants participated as an adventure. They did it to explore, how it feels to ‘just’ take part in a clinical trial [35]. This shows that people have various reasons for volunteering to be a research study participant.

### Strengths and limitations

Our study has some limitations, the main one being that interviewees and discussants were asked about anticipated participation. As such, they were not able to speak from experience of being a HIC which, as shown in Kenya [11], can change perceptions and expectations through the course of HIC participation. Also, we asked well-educated individuals rather than community members for their perceptions on HIC, perceived risks, and benefits. We targeted the educated community because of the novelty of the concept in LMICs and we sought to explore and understand how effectively communicate the concept. Given community safety concerns and role in individual decision-making, communal views including from those less educated will be important. Nonetheless, our study provides insights from individuals who likely comprehend HIC and, aligns with recommendations in literature to first recruit students [11, 26]. Students are most likely to represent healthy volunteers for HIC and, in countries where HIC is yet to be introduced, may more accurately convey their experience and decrease the risk of circulation of misinformation. However, we are alive to the fact that, it is possible that responses given by some students may have been due to social desirability bias, in that they chose to give desirable responses. Finally, given that the study was qualitative, the findings are not transferable but may apply in settings with the similar characteristics.

## Conclusion

This study on views of would-be participants for HIC in Zambia provides the basis to prepare the ground for implementation of HIC that meet participant expectations without compromising research ethics or findings as a result of getting unwanted or unwarranted results due to being uninformed of the research needs of participants. HIC as and when conducted, should consider both community and individual benefits while minimizing the risks in that particular country context. Furthermore, understanding the reasons for participation is vital for the process of providing informed and adequate consent processes. It is also imperative to continue engaging with the wider community for a broader perspective.

What’s more, recruitment of participants in HIC should not only consider educated participants but researchers should endeavor to make information on HIC clear and simple for lay would be participants. It must also be noted that wide consultations with would be participants’ significant others may be crucial for the smooth running and in some cases to aid with participants recruitment and retention.

Besides, LMICs need to build facilities that are comfortable (not hospital like) and appropriate for HIC in-patient studies, as a way to minimize the discomforts that come with being in a hospital set-up.

## Supporting information

S1 Codebook (DOCX)

S1 Checklist (PDF)

## Abbreviations

FGD: Focus Group Discussion
HIC: Human Infection Challenge Studies
HIV: Human Immune deficiency
IDI: In-depth Interview
LMICs: Low and Middle Income countries
RAs: Research Assistants
UTH: University Teaching Hospital
