# Supplementary material for: Exploring willingness to participate in future Human Infection Studies in Lusaka, Zambia: A nested qualitative exploratory study
Source: PLoS One. 2021 Jul 9;16(7):e0254278. doi: 10.1371/journal.pone.0254278 (PMC8270142; doi:10.1371/journal.pone.0254278)
Supplement: S1 Codebook — (DOCX) [file pone.0254278.s001.docx]

**HIC typhoid Code book**

| **Data/quote** | **Code** | **Code definition** |
| --- | --- | --- |
| *Not much but I can say it is bacterial disease, waterborne as well caused by a bacteria called Salmonella Typhi* ***(p3FGD1****).*  *Like P3 said in his introduction he said by consuming contaminated food, water and by getting into contact by someone who is infected by the same (****p9FGD4).*** | Knowledge on typhoid | Applies to all statements on what typhoid is. Can be applied to knowing and not knowing anything about the disease |
| *P8: My perception about people who have typhoid looking at the adverts that we see they tell us keep your environments clean if you don’t then what not so the notion that I had before this was people who get typhoid are dirty. That’s the perception that I had that they are not clean they don’t take care of the environment that’s the perception I had before now* ***(p8FGD3).*** | Perception/thoughts of typhoid patients | What the community says about someone who has suffered from typhoid or how they perceive one who suffers from typhoid |
| *I personally experienced it and it almost took my life because I was being given a malaria treatment because you know like I had similar symptoms like fever …****(p4FGD3)*** | Family typhoid experience | Answers the question has anyone you know or related to suffered from typhoid? |
| *“I will be very honest with you the only person who was really close to him was his biological children and wife till he died. You know this African setup it takes someone to really care for someone till they die to show that affection and love. But during his sickness it was his wife and children who took care for him”* ***(P10FGD4)*** | Care for a typhoid patient | Describes how one was cared for at the time they had typhoid |
| *“I think the main focus is coming up with vaccines and they are looking at coming up with vaccines that will help people in the future, so it is more depicting like don’t just focus now but also in the future for people that might get infected in the near future for prevention through using the vaccine”* ***(p5FGD1).*** | Knowledge on HIC | Explains what a participant knows about Human Infection Challenge Studies |
| *P: mmm, that one depends on you, like what you can explain to me because you have to tell me what it will warrant for, yes, all the side effects.If you tell me that they are too severe I can’t but if there are higher chances of me surviving than me dying, I can participate* ***(IDIp2).*** | Participating in HIC | Answers the question of whether or not one would participate in a HIC study |
| *…what I mean is if there are people dying and there is really need to save those people, I have no problem but if I have to do with me because of my poverty status, then I has to be a huge sum of money that can move me as I said that since I’m a student I need money for seven years and then someone comes and say I will give you a hundred thousand which I know will cover those three or four years that I’m remaining with then I can be like okay I can do it so that my parents do have to suffer any more (laugh)****(IDIp3)*** | Motivation for participation | Refers to reasons why one would participate in a HIC study |
| *That is what you said get the standard treatment the one that is available on the market_ what if it not available – then you die* ***(p12FGD3).*** | Fears and concerns of participation | Issues that may hinder one in participating in a HIC study |
| *aah, no one* ***(IDIp11).***  *… I will definitely need to tell my parents they will not be happy, I think my mom will more understanding than my dad but I can convince him that for the seek of science and I have to convince him that I will definitely be safe, yeah, I would consult not I need a go ahead but atleast I need then to understand, I need then to be on my side, so I would convince them that way****(IDIp13)*** | Consultation | To do with whom one chooses to consult in order to take part in a HIC study |
| *I feel I wouldn’t love to be really isolated from my family because I would need support in times like this and I need to be around them so that I can feel self and not more than two weeks****(FGD2)***  *Not more than 1 week _ not more than 1 week _ yes because you have also a life besides that. So if they take you on more than 2 weeks then that can be too much.* | Isolation | Comments on how long a participant would be willing to be isolated and leave the family/loved ones |
| *…as long as it is not a hospital environment where I feel like I’m sick but atleast like they make me feel like I’m home, doing things I love to do, for others they can love to be playing tennis so atleast give someone something that they do(****IDIp3).*** | Kind of research facility | The kind of facility a participant expresses as desirable |
| *Okay so about them injecting a person with a vaccine and then later on injecting them with an infection uhhm in instances where the vaccine doesn’t work and the person falls ill, what are they going to use to treat a person in that they are creating a vaccine to treat that same infection. So what do they use to treat a person if the vaccine they injected them at first doesn’t work? That is my question* ***(p8FGD3).***  *P4: not just that also starvation my dear coz when his gone for that process it means no bedroom issues you know what I mean? So those are some of the …* | Fears and concerns of participation | Refers to questions and thoughts of doubt expressed by the participants |
| *“First they should try it on animals or that…. ,may be it work out with an error and if it will not work* ***(FGD3).***  *Yes because you might find that there just junior trainees – we don’t want that because this is life we are dealing with – so we want qualified people who can handle the issues at hand and I would want them to talk to me one on one – to take me through each step you know it is my life”****(FGD3)*** | Expectations/Recommendations | Expressions of what should be the case or should be done |
| *I think just like my grand mother if you have a running stomach, she will give you ashes to drink and it stops the stomach from hurting so I think so I think ashes, tembusha (aloe Vera) and what else there is this…. Just those.. So you boil aloe Vera then you drink the water…****(p1FGD3)*** | Traditional remedies | Traditional methods that can be used to treat typhoid |
| *I think as a husband the best thing to do is to learn from her and hear what is involved into doing it before I react to that situation. Because in as much as we know sometimes our female folk have that trend to say that it is really important to sit and discuss before you react it be a very good cause, but I would generally want to sit down and ask and learn from her and really find out what prompted her to participate in the Human Infection Challenge* ***(FGD5)*** | Perceptions on family members participating | Responses to do with whether or not one would allow a family member to participate in a HIC study |
| *P1: I think if the vaccine works they try it on me then it works then they will make more, they will bring it to the community they are going to benefit because there will be less typhoid* ***(FGD4)*** | Benefits for the community | Refers to all that was attributed as benefits for the community if one participated in a HIC study |
