# Supplementary material for: Exploring willingness to participate in future Human Infection Studies in Lusaka, Zambia: A nested qualitative exploratory study
Source: PLoS One. 2021 Jul 9;16(7):e0254278. doi: 10.1371/journal.pone.0254278 (PMC8270142; doi:10.1371/journal.pone.0254278)
Supplement: S1 Checklist — (PDF) [file pone.0254278.s002.pdf]

Standards for Reporting Qualitative Research (SRQR)

Please indicate in which section, figure or table each item has been reported in your manuscript. If you feel that an item does not apply to your manuscript, please enter N/A.

For more information about the SRQR guidelines, please see O'Brien *et al.*, 2014: https://doi.org/10.1097/ACM.0000000000000388

No.	Item	Description	Section
Title and abstract			
1	Title	Concise description of the nature and topic of the study. Identifying the study as qualitative or indicating the approach (e.g., ethnography, grounded theory) or data collection methods (e.g., interview, focus group) is recommended	Pg1
2	Abstract	Summary of key elements of the study using the abstract format of the intended publication; typically includes background, purpose, methods, results, and conclusions	Pg1
Introduction			
3	Problem formulation	Description and significance of the problem/phenomenon studied; review of relevant theory and empirical work; problem statement	Pg3
4	Purpose or research question	Purpose of the study and specific objectives or questions	Pg 1, 4,5
Methods			
5	Qualitative approach and research paradigm	Qualitative approach (e.g., ethnography, grounded theory, case study, phenomenology, narrative research) and guiding theory if appropriate; identifying the research paradigm (e.g., postpositivist, constructivist/ interpretivist) is also recommended; rationale*	1
6	Researcher characteristics and reflexivity	Researchers' characteristics that may influence the research, including personal attributes, qualifications/experience, relationship with participants, assumptions, and/or presuppositions; potential or actual interaction between researchers' characteristics and the research questions, approach, methods, results, and/or transferability	N/A
7	Context	Setting/site and salient contextual factors; rationale*	Pgs 4,5
8	Sampling strategy	How and why research participants, documents, or events were selected; criteria for deciding when no further sampling was necessary (e.g., sampling saturation); rationale*	Pgs 6,7
9	Ethical issues pertaining to human subjects	Documentation of approval by an appropriate ethics review board and participant consent, or explanation for lack thereof; other confidentiality and data security issues	Pg 13
10	Data collection methods	Types of data collected; details of data collection procedures including (as appropriate) start and stop dates of data collection and analysis, iterative process, triangulation of sources/methods, and modification of procedures in response to evolving study findings; rationale*	Pgs 7,8
11	Data collection instruments and technologies	Description of instruments (e.g., interview guides, questionnaires) and devices (e.g., audio recorders) used for data collection; if/how the instrument(s) changed over the course of the study	Pgs 7
12	Units of study	Number and relevant characteristics of participants, documents, or events included in the study; level of participation (could be reported in results)	Pgs 5,6
13	Data processing	Methods for processing data prior to and during analysis, including transcription, data entry, data management and security, verification of data integrity, data coding, and anonymization/deidentification of excerpts	Pgs 5,8

14	Data analysis	Process by which inferences, themes, etc., were identified and developed, including the researchers involved in data analysis; usually references a specific paradigm or approach; rationale*	Pgs 10-13
15	Techniques to enhance trustworthiness	Techniques to enhance trustworthiness and credibility of data analysis (e.g., member checking, audit trail, triangulation); rationale*	Pg 10
Results/findings			
16	Synthesis and interpretation	Main findings (e.g., interpretations, inferences, and themes); might include development of a theory or model, or integration with prior research or theory	Pgs 13-24
17	Links to empirical data	Evidence (e.g., quotes, field notes, text excerpts, photographs) to substantiate analytic findings	Pgs 14-24
Discussion			
18	Integration with prior work, implications, transferability, and contribution(s) to the field	Short summary of main findings; explanation of how findings and conclusions connect to, support, elaborate on, or challenge conclusions of earlier scholarship; discussion of scope of application/ generalizability; identification of unique contribution(s) to scholarship in a discipline or field	Pgs 24-26
19	Limitations	Trustworthiness and limitations of findings	Pgs 27,28
Other			
20	Conflicts of interest	Potential sources of influence or perceived influence on study conduct and conclusions; how these were managed	Pg 29
21	Funding	Sources of funding and other support; role of funders in data collection, interpretation, and reporting	Pg 30

*The rationale should briefly discuss the justification for choosing that theory, approach, method, or technique rather than other options available, the assumptions and limitations implicit in those choices, and how those choices influence study conclusions and transferability. As appropriate, the rationale for several items might be discussed together.

When submitting your manuscript via the online submission form, please upload the completed checklist as a Figure/supplementary file.

If you would like this checklist to be included alongside your article, we ask that you upload the completed checklist to an online repository and include the guideline type, name of the repository, DOI and license in the *Data availability* section of your manuscript.

Developed from: Bridget C. O'Brien, Ilene B. Harris, Thomas J. Beckman, Darcy A. Reed, David A. Cook. Standards for Reporting Qualitative Research: A Synthesis of Recommendations. Academic Medicine, Volume 89, Issue 9, September 2014, Pages 1245-1251. https://doi.org/10.1097/ACM.0000000000000388
